# Pathological 25 kDa C-Terminal Fragments of TDP-43 Are Present in Lymphoblastoid Cell Lines and Extracellular Vesicles from Patients Affected by Frontotemporal Lobar Degeneration and Neuronal Ceroidolipofuscinosis Carrying a *GRN* Mutation

**DOI:** 10.3390/ijms232213753

**Published:** 2022-11-09

**Authors:** Sara Cimini, Sonia Bellini, Claudia Saraceno, Luisa Benussi, Roberta Ghidoni, Silvia Clara Giliani, Gianfranco Puoti, Laura Canafoglia, Giorgio Giaccone, Giacomina Rossi

**Affiliations:** 1 Unit of Neurology V-Neuropathology, Fondazione IRCCS Istituto Neurologico Carlo Besta, 20133 Milan, Italy; 2 Molecular Markers Laboratory, IRCCS Istituto Centro San Giovanni di Dio Fatebenefratelli, 25125 Brescia, Italy; 3 Department of Molecular and Translational Medicine, “Angelo Nocivelli” Institute for Molecular Medicine, University of Brescia, ASST Spedali Civili, 25123 Brescia, Italy; 4 Department of Advanced Medical and Surgical Sciences, University of Campania “L. Vanvitelli”, 80131 Naples, Italy; 5 Integrated Diagnostics for Epilepsy, Fondazione IRCCS Istituto Neurologico Carlo Besta, 20133 Milan, Italy

**Keywords:** TDP-43, *GRN*, mutation, FTLD, NCL, EVs, LCLs

## Abstract

Frontotemporal lobar degeneration (FTLD) is a complex disease, characterized by progressive degeneration of frontal and temporal lobes. Mutations in progranulin (*GRN*) gene have been found in up to 50% of patients with familial FTLD. Abnormal deposits of post-translationally-modified TAR DNA-binding protein of 43 kDa (TDP-43) represent one of the main hallmarks of the brain pathology. To investigate in peripheral cells the presence of the different TDP-43 forms, especially the toxic 25 kDa fragments, we analyzed lymphoblastoid cell lines (LCLs) and the derived extracellular vesicles (EVs) from patients carrying a *GRN* mutation, together with wild-type (WT) healthy controls. After characterizing EV sizes and concentrations by nanoparticle tracking analysis, we investigated the levels of different forms of the TDP-43 protein in LCLs and respective EVs by Western blot. Our results showed a trend of concentration decreasing in EVs derived from *GRN*-mutated LCLs, although not reaching statistical significance. A general increase in p-TDP-43 levels in *GRN*-mutated LCLs and EVs was observed. In particular, the toxic 25 kDa fragments of p-TDP-43 were only present in GRN-mutated LCLs and were absent in the WT controls. Furthermore, these fragments appeared to be more concentrated in EVs than in LCLs, suggesting a relevant role of EVs in spreading pathological molecules between cells.

## 1. Introduction

Frontotemporal lobar degeneration (FTLD) is a complex disease, characterized by progressive degeneration of frontal and temporal lobes and extensive neuroinflammation, which manifests with a range of clinical disorders and inevitably leads to death. FTLD is the second most common cause of dementia after Alzheimer’s disease (AD) under the age of 65 [1,2]. About 40% of cases of FTLD have a positive family history, and the main causative genes linked to the disease are microtubule-associated protein tau (*MAPT*), chromosome 9 open reading frame 72 (*C9ORF72*), and progranulin (*GRN*), with many other genes being less frequent [2].

*GRN* mutations have been found in about 10% of patients with sporadic FTLD and in up to 50% of patients with familial FTLD [3,4]. Progranulin (PGRN) is a widely expressed secreted growth factor which plays a role in multiple processes, including development, wound repair, and inflammation [5,6]. In the central nervous system, PGRN modulates inflammation and acts as a neurotrophic and neuroprotective factor [7,8,9,10]. Most *GRN* mutations areloss of function, leading to mutated mRNA degradation and to a following condition of haploinsufficiency [11,12]. While heterozygous *GRN* mutations cause FTLD, homozygous *GRN* mutations cause neuronal ceroid lipofuscinosis (NCL), a lysosomal storage disorder [13], although a few homozygous cases have shown FTLD phenotype [14].

The main neuropathological hallmarks of *GRN*-associated FTLD include neuronal loss, glia hyperproliferation and inflammation, lysosomal dysfunction, and, in particular, abnormal deposits of TAR DNA-binding protein of 43 kDa (TDP-43) in neuronal cells [15,16,17]. Nuclear factor TDP-43 is a multifunctional RNA binding protein that plays a role in many cellular processes, such as transcription, RNA splicing, stability, transport, and translation [18]. TDP-43 presents a predominantly nuclear localization, although it can shuttle between the nucleus and cytosol. In the pathological aggregates found in brains of patients with FTLD, TDP-43 undergoes different kinds of post-translational modifications such as ubiquitination, phosphorylation at Ser403/Ser404 or Ser409/Ser410, and abnormal cleavage to generate C-terminal fragments (CTFs) of ~25 kDa and ~35 kDa, which are more prone to forming aggregates than the full-length (FL) molecule [17,19,20,21,22,23,24].

In cellular models of TDP-43 proteinopathies and in human brain samples from FTLD, FL TDP-43 and its CTFs are released via exosomes [25,26]. An enrichment of exosomal TDP-43 CTFs has also been shown in cerebrospinal fluid derived from FTLD patients [27]. Exosomes are the smallest extracellular vesicles (EVs) (30–100 nm diameter) and are delimited by a lipid bilayer and secreted by most cell types into the extracellular environment. EVs carry different kinds of biological molecules, such as nucleic acids, lipids, and proteins [28]. These molecules can accomplish physiological communication between cells but can also transmit toxicity if they are pathologically altered as originated in a disease environment. EVs are key players in physiological processes such as synaptic plasticity and myelination maintenance, whereas in neurodegenerative diseases, such as Alzheimer’s disease, Parkinson’s disease, and FTLD, EVs may play roles in the formation, spreading, and clearance of toxic protein aggregates [29].

In this work, we investigated the presence of TDP-43 and its phosphorylated/pathological forms in peripheral cells from FTLD and NCL patients carrying a *GRN* mutation and wild-type (WT) healthy control subjects. We analyzed both lymphoblastoid cell lines (LCLs) and the derived EVs, searching for pathological hallmarks. We observed the presence of phosphorylated 25 kDa CTFs of TDP-43 in cells originated from subjects carrying a *GRN* mutation, while these fragments were absent in WT control cell lines. Furthermore, we confirmed the same findings in the EVs, suggesting that EVs actually reflect the pathological status of their originating cells.

## 2. Results

### 2.1. PGRN Expression in GRN-Mutated Lymphoblastoid Cell Lines

**Figure 1:**
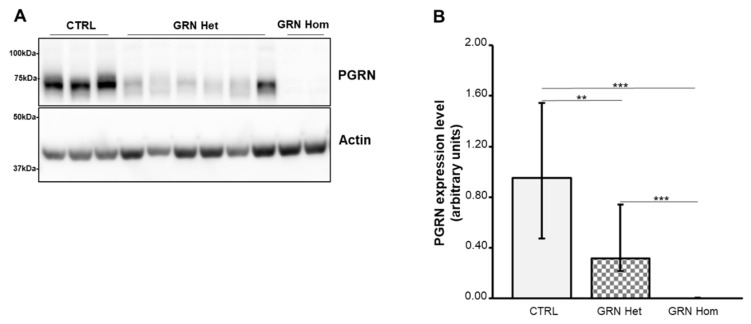
PGRN content in LCLs from WT controls and heterozygous and homozygous *GRN* mutation carriers. (**A**) A representative Western blot is shown. (**B**) Densitometric analysis of PGRN. Actin was used as the loading control. The graph represents medians with interquartile ranges (25–75%). Data are from six independent experiments in three WT and nine *GRN*-mutated LCLs. Statistical analysis by Mann–Whitney test, ** *p* < 0.01; *** *p* < 0.001. Het = heterozygous; Hom = homozygous.

Nearly all pathogenic *GRN* mutations result in *null* alleles, leading to a reduction of PGRN (haploinsufficiency). PGRN expression was evaluated in LCLs from three healthy WT subjects and nine patients carrying *GRN null* mutations (heterozygous mutations: Thr272SerfsX10, Cys149LeufsX10, Gln341X, IVS1-2A>G splicing; homozygous mutation Thr272SerfsX10). As expected, PGRN levels were significantly reduced in heterozygous *GRN* mutation carriers (*p* = 0.0011), while the protein was absent in homozygous carriers (*p* = 0.0002) (Figure 1).

### 2.2. TDP-43 Expression in GRN-Mutated Lymphoblastoid Cell Lines

As it is known that pathological TDP-43 is abnormally phosphorylated at Ser409/Ser410 residues and cleaved to generate CTFs migrating at ~25 kDa, which are more prone to phosphorylation than FL protein, we examined all these forms of TDP-43 in LCLs from heterozygous and homozygous *GRN* mutation carriers. Western blots were performed with antibodies recognizing TDP-43 or TDP-43 phosphorylated at residues Ser409/Ser410 (p-TDP-43).

**Figure 2:**
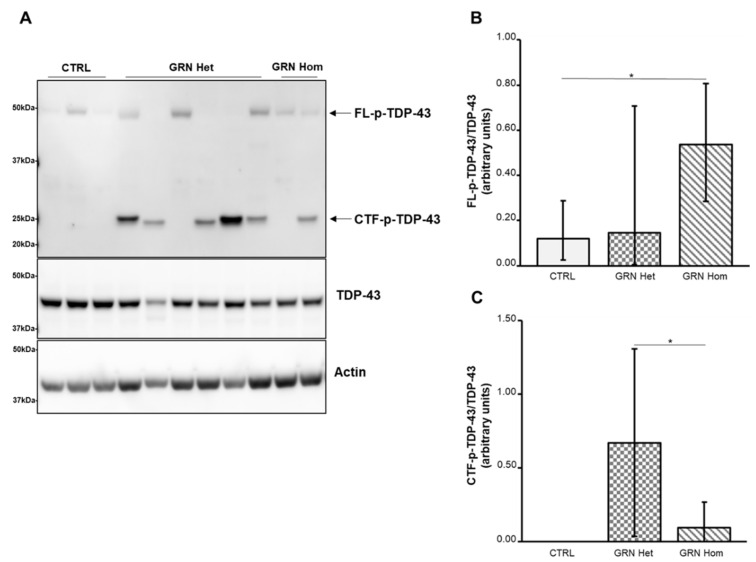
TDP-43 content in LCLs from WT controls and heterozygous and homozygous *GRN* mutation carriers. (**A**) A representative Western blot showing TDP-43 phosphorylated at Ser409/Ser410 residues (upper panel) or total TDP-43 (middle panel). (**B**) Densitometric analysis of FL p-TDP-43 normalized to the respective TDP-43. The graph represents median with interquartile ranges (25–75%). Statistical analysis by Mann–Whitney test, * *p* < 0.05. (**C**) Densitometric analysis of the 25 kDa CTFs p-TDP-43 normalized to the respective TDP-43. The graph represents media ± SD. Student’s *t*-test, * *p* < 0.05. Data are from six independent experiments in three WT and nine *GRN*-mutated LCLs. Het = heterozygous; Hom = homozygous.

We observed an overall increase in p-TDP-43 levels in *GRN*-mutated LCLs (Figure 2): while FL p-TDP-43 was significantly higher, with respect to the controls, only in *GRN* homozygous LCLs (*p* = 0.0136, Figure 2B), showing a variable expression in *GRN* heterozygous LCLs, the 25 kDa phosphorylated CTFs (CTFs p-TDP-43) were exclusively detected in *GRN*-mutated LCLs (Figure 2C), although not in all of them, being absent in WT controls. The 25 kDa p-CTFs contents were significantly higher in *GRN* heterozygous LCLs compared to *GRN* homozygous LCLs (*p* = 0.0272).

### 2.3. TDP-43 Expression in Extracellular Vesicles from GRN-Mutated Lymphoblastoid Cell Lines

**Figure 3:**
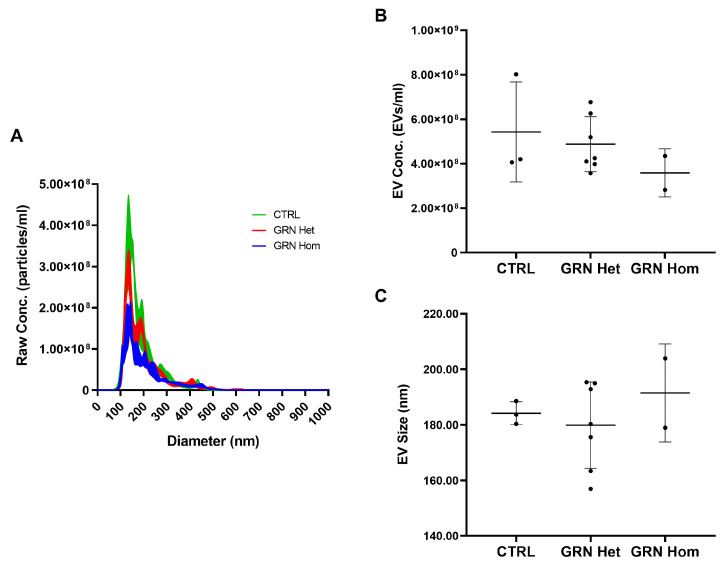
EV concentrations and sizes in LCLs from WT controls and heterozygous and homozygous *GRN* mutation carriers. (**A**) Representative spectra from NTA of CTRL (green), *GRN* Het (red), and *GRN* Hom (blue) EVs isolated from LCL-conditioned media. (**B**) EV concentration (EVs/mL) and (**C**) EV size (nm) in LCL-conditioned media from WT controls and *GRN* mutation carriers. Graphs represent mean ± SD. Statistical analysis by one-way ANOVA with Bonferroni post-hoc tests. Het = heterozygous; Hom = homozygous.

As it has been demonstrated that the EVs carry different kinds of biological molecules, including proteins, which can either accomplish physiological communication between cells or transmit toxicity if they are pathologically altered, we examined the contents of EVs from *GRN*-mutated LCLs in order to assess the presence of pathological forms of TDP-43. We isolated the EVs from conditioned media of control and *GRN*-mutated LCLs and firstly analyzed them by nanoparticle tracking analysis (NTA) to evaluate EV concentrations (EVs/mL) and average sizes (nm). Only a trend toward a decrease in EV concentration in *GRN*-mutated LCLs compared to controls was observed, even if not reaching statistical significance (Figure 3A,B) (mean ± SD: CTRL 5.43 × 10^8^ ± 2.25 × 10^8^, *GRN* Het 4.87 × 10^8^ ± 1.23 × 10^8^, *GRN* Hom 3.59 × 10^8^ ± 1.08 × 10^8^; *p* = 0.433, one-way ANOVA test). No statistically significant differences in EV size were observed among the investigated groups (Figure 3A,C) (mean ± SD: CTRL 184.23 ± 4.13, *GRN* Het 179.94 ± 15.55, *GRN* Hom 191.50 ± 17.65; *p* = 0.602, one-way ANOVA test).

**Figure 4:**
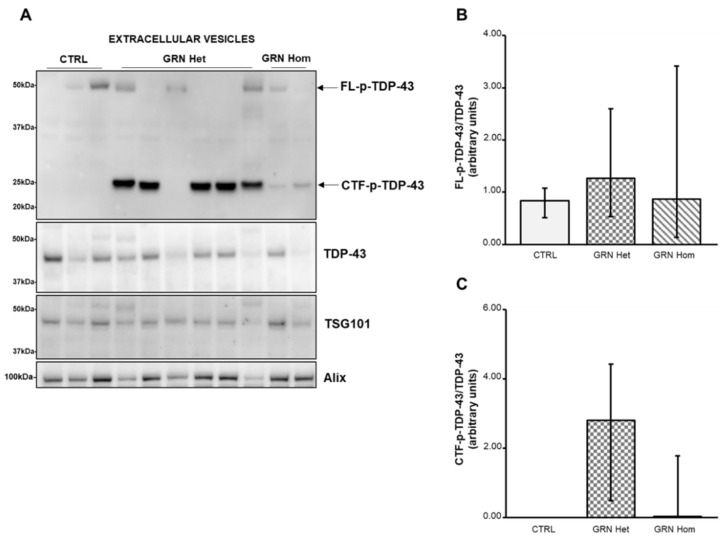
TDP-43 content in EVs isolated from LCLs from WT controls and heterozygous and homozygous *GRN* mutation carriers. (**A**) A representative Western blot showing TDP-43 phosphorylated at Ser409/Ser410 residues (upper panel) or total TDP-43 (middle panel). TSG101 and Alix are also shown. (**B**) Densitometric analysis of FL p-TDP-43 normalized to the respective TDP-43. (**C**) Densitometric analysis of the 25 kDa CTFs p-TDP-43 normalized to the respective TDP-43. The graph represents median with interquartile ranges (25–75%). Data are from six independent experiments in three WT and nine *GRN*-mutated LCLs. Statistical analysis by Mann–Whitney test, Het = heterozygous; Hom = homozygous.

Then, we performed Western blot analysis to investigate the forms of TDP-43 carried by the EVs (Figure 4). The FL p-TDP-43 showed a variable expression both in WT and in *GRN*-mutated LCLs, with no significant difference (Figure 4B). On the contrary, the 25 kDa CTFs p-TDP-43 were only present in *GRN*-mutated LCLs (Figure 4C), although not in all of them, being absent in WT controls, reflecting the findings from the respective LCLs.

TSG101 and Alix proteins were revealed as EVs markers by their respective antibodies.

## 3. Discussion

FTLD linked to *GRN* mutations is pathologically characterized by the presence of abnormal deposits of TDP-43 in brain neurons [16,17]. This RNA-binding protein is mostly mislocalized from the nucleus to cytoplasm, where it becomes insoluble and aggregates. Further, some rare intranuclear lentiform aggregates are also found. In addition, TDP-43 is subjected to multiple post-transductional modifications, such as ubiquitination, phosphorylation, and cleavage [30]. CTFs of TDP-43 have been demonstrated to be more prone to aggregate and more toxic than the FL protein. Cells transfected with the 25 kDa TDP-43 CTF showed clear toxicity with high lactate dehydrogenase levels, fragmented nuclei, and activated caspase 3, indicative of apoptosis [24]. TDP-43 CTFs fragments around 25 kDa expressed in cultured cells caused the formation of abnormal aggregates similar to those found in the brains of patients [22]. Animal models expressing the 25 kDa TDP-43 CTF show evidence that high levels of this fragment are sufficient to cause behavioral deficits and reduce the function of autophagy and proteasomes [31].

We have a collection of LCLs derived from patients carrying a *GRN* mutation, both heterozygous, affected by FTLD, and homozygous, affected by NCL. We hypothesized that peripheral cells may reflect the pathological changes present in brain tissue of these patients and decided to investigate the main protein involved, TDP-43, in its different forms, especially the 25 kDa fragments, both in LCLs and their EVs. To date, no such data on peripheral cells from *GRN*-linked FTLD/NCL patients have been reported, while there are some data on the plasma, concerning the FL p-TDP-43. Plasma FL p-TDP-43 was generally higher in FTLD with pathological TDP-43 changes (included 6 *GRN*-mutated patients), although this did not reach statistical significance [32]. In another study, plasma FL p-TDP-43 levels were significantly increased in subjects carrying a C9orf72 repeat expansion (*n* = 10) or *GRN* mutations (*n* = 5) compared with sporadic FTLD subjects (*n* = 51) and controls (*n* = 22) [33]. An additional study showed an enrichment of the 35 kDa fragment in plasma EVs from sporadic FTLD patients. [34].

In our LCLs, TDP-43 levels were similar among all genotypes, including WT, heterozygous and homozygous, both in LCLs and EVs. This suggests that protein transcription levels and stability do not differ significantly, even in presence of the mutation. Similar levels of TDP-43 were also reported in LCLs from ALS and AD cases compared to healthy controls [35,36]. On the contrary, p-TDP-43 showed some differences between WT and mutated LCLs. FL p-TDP-43 levels were significantly higher in *GRN* homozygous LCLs than in WT LCLs, while the situation was more variable in *GRN* heterozygous LCLs, as some of them did not display FL p-TDP-43. However, concerning the CTFs p-TDP-43 of about 25 kDa, the differences between WT and mutated LCLs were striking. In fact, CTFs p-TDP-43 were not detected in WT LCLs, whereas in all but one *GRN* heterozygous LCLs and one *GRN* homozygous LCL they were present at high expression level. Interestingly, in the cases where CTFs p-TDP-43 were absent, FL p-TDP-43 was present, indicating that at least one abnormal form of TDP-43 is present anyway, perhaps not (yet) truncated to CTFs.

As for EVs, while in plasma and in human primary fibroblasts from *GRN* patients, a significant concentration decrease of EVs was described [37,38], in our EVs from *GRN*-mutated LCLs only a trend towards concentration decrease was observed, not reaching statistical significance. In EVs, the level of FL p-TDP-43 did not show significant difference among genotypes, but the level of 25 kDa CTFs p-TDP-43 confirmed the situation of the respective LCLs: CTFs p-TDP-43 were absent in WT EVs, very abundant in *GRN* heterozygous EVs and also present, at lower levels, in *GRN* homozygous EVs. Comparing the ratio CTFs p-TDP-43/TDP-43 in LCLs to the ratio in EVs, the amounts of CTFs p-TDP-43 resulted increased in heterozygous EVs, that is the EVs appeared to concentrate these toxic fragments. This finding makes the EVs particularly relevant and critical in their role of communication between cells, as previous studies have demonstrated that TDP-43 aggregates can be transferred from cell to cell via exosomes [25].

The finding that in *GRN* homozygous LCLs and EVs the CTFs p-TDP-43 were at lower levels than in *GRN* heterozygous is intriguing. Homozygous *GRN* mutations are associated with NCL, a disease that shares some pathological features with FTLD but that is also very different. In a recent post-mortem analysis of a human brain carrying a homozygous *GRN* mutation, only a very mild TDP-43 pathology was described, without any positive detection of aggregated forms, while lysosomal storage disorder was overwhelming [14]. Our data are in agreement with these findings, indicating that TDP-43 pathology is mostly exacerbated in *GRN* heterozygous FTLD context.

As EVs are released into the extracellular space and can be isolated from a number of body fluids, including blood, CSF and urine, detecting molecules associated with these vesicles may have diagnostic, prognostic, and disease monitoring potential. We think that the toxic 25 kDa CTFs p-TDP-43 detected in LCLs and their EVs may have this potential, as they are derived from peripheral cells. As a future direction, detection of these fragments in plasma or plasma EVs may be planned.

## 4. Materials and Methods

### 4.1. Subjects

Following genetic analysis for diagnostic purposes in the dementia field, we disclosed some patients carrying a *GRN* mutation. After acquiring informed consent for research purposes from these patients and control subjects, we obtained peripheral blood lymphocytes, from which LCLs were produced using the Epstein–Barr virus infection, according to standard procedures.

LCLs from three WT healthy age-matched control subjects and nine patients carrying a *GRN* (RefSeq NM_002087.4) mutation (seven patients carrying the heterozygous mutations Thr272SerfsX10, Cys149LeufsX10, Gln341X, IVS1-2A>G; two patients carrying the homozygous mutation Thr272SerfsX10) were analyzed in this study.

### 4.2. Cell Cultures

LCLs were cultured in suspension in T25 flasks in Optimem medium (Gibco, Life Technologies Limited, Paisley, UK) containing 10% fetal bovine serum (GE Healthcare Life Sciences Hyclone Laboratories, South Logan, UT, USA) and 2% penicillin/streptomycin (Life Technologies Corporation, Grand Island, NE, USA).

### 4.3. Cellular Lysates

LCLs (10^6^ cells/mL) were seeded in an Optimem medium without FBS for 24 h. The conditioned medium was then collected by centrifugation and used to isolate EVs (see below), while the cells were washed in PBS and lysed in an ice-cold RIPA lysis buffer (50 mM Tris-HCl pH 8, 150 mM NaCl, 5 mM EDTA pH 8, 1% NP-40, 0.5% sodium deoxycholate, 0.1% SDS) containing Protease Inhibitor Cocktail (Roche Diagnostics GmbH, Mannheim, Germany) and Phosphatase Inhibitor Cocktail (Sigma Aldrich, Merk KGaA, Darmstadt, Germany). The cell lysates were kept in ice for 15 min, sonicated in ice, kept for an additional 15 min in ice, centrifuged at 13,000× *g* for 15 min at 4 °C, and then the supernatant was recovered. Protein concentration of extracts was determined by a BCA assay kit (Thermo Fisher Scientific, Waltham, MA, USA).

### 4.4. Extracellular Vesicles Extraction

EVs were isolated from conditioned media of LCLs after 24 h of starvation. EVs were prepared with a Total Exosome Isolation reagent (Invitrogen, Thermo Fisher Scientific, Vilnius, Lithuania) following the manufacturer’s instructions. Briefly, the conditioned cell culture media were centrifuged at 2000× *g* for 30 min to remove cells and debris. Supernatants were mixed with 0.5 volumes of the Total Exosome Isolation reagent and incubated at 4 °C overnight. After incubation, the samples were centrifuged at 10,000× *g* for 1 h at 4 °C. EV-containing pellets were resuspended with 100 µL of 0.2 µM filtered phosphate-buffered saline (PBS) for nanoparticle tracking analysis (NTA) or lysed in an ice-cold RIPA lysis buffer containing Protease Inhibitor Cocktail and Phosphatase Inhibitor Cocktail for Western blot analyses. Protein concentration of extracts was determined by BCA assay kit.

### 4.5. Nanoparticle Tracking Analysis

EVs resuspended in PBS were analyzed with the NanoSight NS300 Instrument (Malvern, Worcestershire, UK). Samples were diluted with filtered PBS to obtain an optimal range of 20–150 particles/frame. For each sample, 5 videos of 60 s were recorded and data were processed using NanoSight NTA Software 3.2. Optimized post-acquisition settings were kept constant during the analysis of all samples. The data obtained included particle concentration (particles/mL) and average size (nm). Raw concentration data (particles/mL) from NTA were normalized to obtain EV concentrations in the LCL-conditioned media.

### 4.6. Western Blot Analyses

Western blot analysis was performed using 25 µg cell lysates and 15 µg EVs lysates. Samples were subjected to polyacrylamide gel electrophoresis using Bolt 4–12% Bis-Tris gels (Thermo Fisher Scientific, Waltham, MA, USA) and transferred onto PVDF membranes (Thermo Fisher Scientific, Waltham, MA, USA). The membranes were incubated overnight at 4 °C with the following primary antibodies: anti-hPGRN (AF2420, 1:500, R&D systems, Minneapolis, MN, USA); anti-TSG101 (ab83, 1:500, Abcam, Cambridge, UK); anti-Alix (SAB4200476, 1:600, Sigma-Aldrich, St. Louis, MO, USA); anti-TDP43 (10782-2-AP, 1:2000, Proteintech, Deansgate Manchester, UK); anti-p-TDP43 (recognising phosphorylation at Ser409/Ser410 residues, MABN14, 1:500, Millipore, Burlington, MA, USA) and anti-Actin (MAB1501R, 1:3000, Millipore, Burlington, MA, USA). TSG101 and Actin were used as loading controls for EVs and cellular extracts, respectively. Horseradish peroxidase-conjugated secondary antibodies (InvitrogenTM, Waltham, MA, USA) were incubated for 1 h at room temperature. Immuno-positive bands were detected by chemiluminescence (GE Healthcare, Milan, Italy) according to the manufacturer’s instructions. Densitometric analysis was performed with QuantityOne software (BIO-RAD).

### 4.7. Statistical Analyses

All statistical analyses were performed using GraphPad Prism Software (San Diego, CA, USA). Data distribution was assessed by D’Agostino and Pearson omnibus normality test. Student’s *t*-test or Mann–Whitney test was used for comparison between two groups of normally or non-normally distributed variables, respectively. One-way ANOVAs, with Bonferroni post-hoc tests, were used for comparisons within the groups of normally distributed variables. Means with standard deviations or medians with interquartile ranges (25–75%) were graphically represented for normally or non-normally distributed variables, respectively. Significance was set at *p* < 0.05.

## Data Availability

The raw data supporting the conclusions of this article are available in the Zenodo Data Repository (10.5281/zenodo.7071685).
